# 

**Published:** 1887-04

**Authors:** 


*Entered at the Post Office, at Austin, Texas, as Second-Class Mail Matter.*

Vol. II. APRIL, 1887.

### DANIEL'S MEDICAL JOURNAL

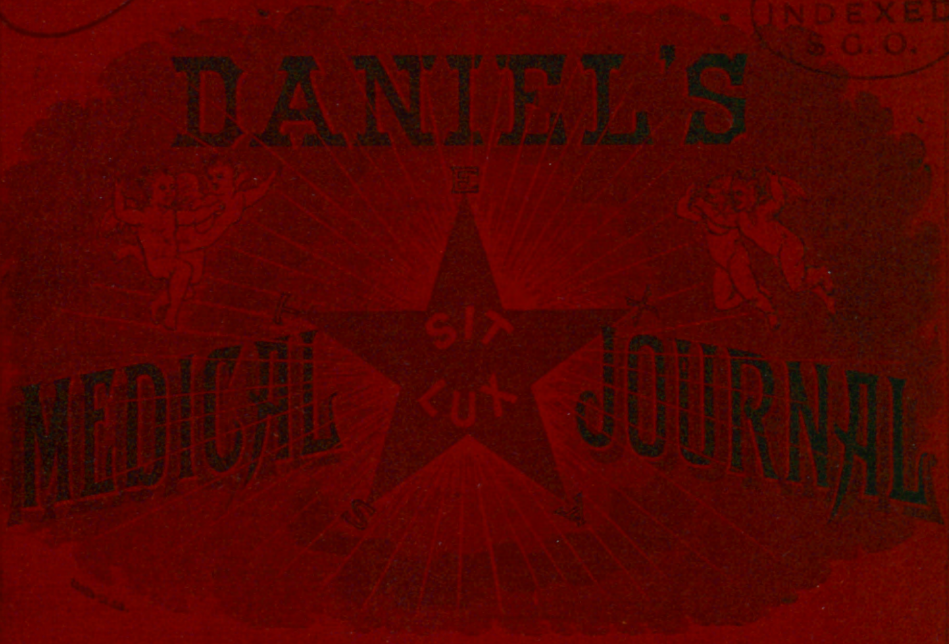

Subscription, $2.00 a Year, in Advance. — Single Copies, Twenty-five Cents.

F. E. DANIEL, M. D., Editor and Publisher, AUSTIN, TEXAS.

